# Dose finding study for unilobar radioembolization using holmium-166 microspheres to improve resectability in patients with HCC: the RALLY protocol

**DOI:** 10.1186/s12885-023-11280-9

**Published:** 2023-08-18

**Authors:** Daan Andel, Marnix G. E. H. Lam, Joep de Bruijne, Maarten L. J. Smits, Arthur J. A. T. Braat, Adriaan Moelker, Erik Vegt, Simeon J. S. Ruiter, Walter Noordzij, Gianluca Grazi, Giulio E. Vallati, Roel J. Bennink, Otto M. van Delden, Onno W. Kranenburg, Jan N. M. Ijzermans, Maarten W. Nijkamp, Joris I. Erdmann, Rosa Sciuto, Jeroen Hagendoorn, Inne H. M. Borel Rinkes

**Affiliations:** 1 Department of Surgical Oncology, University Medical Center Utrecht, Cancer Center, PO BOX 85500, 3508 GA Utrecht, The Netherlands; 2 Department of Radiology and Nuclear Medicine, University Medical Center Utrecht, Cancer Center, Utrecht, The Netherlands; 3 Department Gastroenterology and Hepatology, University Medical Center Utrecht, Utrecht, the Netherlands; 4 Department of Radiology and Nuclear Medicine, Erasmus MC University Medical Center, Rotterdam, The Netherlands; 5 Department of HPB & Liver Transplantation, University Medical Center Groningen, Groningen, The Netherlands; 6 Department of Nuclear Medicine and Molecular Imaging, University Medical Center Groningen, Groningen, The Netherlands; 7 Hepatopancreatobiliary Surgery, IRCCS - Regina Elena National Cancer Institute, Rome, Italy; 8 Interventional Radiology, IRCCS - Regina Elena National Cancer Institute, Rome, Italy; 9 Department of Radiology and Nuclear Medicine, Cancer Center, Amsterdam UMC, Location University of Amsterdam, Amsterdam, The Netherlands; 10 Department of Surgery, Erasmus MC University Medical Center, Rotterdam, The Netherlands; 11 Department of Surgery, Cancer Center, Amsterdam UMC, Location Vrije Universiteit Amsterdam, Amsterdam, The Netherlands; 12 Nuclear Medicine, IRCCS - Regina Elena National Cancer Institute, Rome, Italy

**Keywords:** Radiation lobectomy, Radioembolization, Holmium-166, ^166^Ho, Hepatocellular carcinoma, Unilobar radioembolization

## Abstract

**Background:**

High dose unilobar radioembolization (also termed ‘radiation lobectomy’)—the transarterial unilobar infusion of radioactive microspheres as a means of controlling tumour growth while concomitantly inducing future liver remnant hypertrophy—has recently gained interest as induction strategy for surgical resection. Prospective studies on the safety and efficacy of the unilobar radioembolization-surgery treatment algorithm are lacking. The RALLY study aims to assess the safety and toxicity profile of holmium-166 unilobar radioembolization in patients with hepatocellular carcinoma ineligible for surgery due to insufficiency of the future liver remnant.

**Methods:**

The RALLY study is a multicenter, interventional, non-randomized, open-label, non-comparative safety study. Patients with hepatocellular carcinoma who are considered ineligible for surgery due to insufficiency of the future liver remnant (< 2.7%/min/m^2^ on hepatobiliary iminodiacetic acid scan will be included. A classical 3 + 3 dose escalation model will be used, enrolling three to six patients in each cohort. The primary objective is to determine the maximum tolerated treated non-tumourous liver-absorbed dose (cohorts of 50, 60, 70 and 80 Gy). Secondary objectives are to evaluate dose–response relationships, to establish the safety and feasibility of surgical resection following unilobar radioembolization, to assess quality of life, and to generate a biobank.

**Discussion:**

This will be the first clinical study to assess the unilobar radioembolization-surgery treatment algorithm and may serve as a stepping stone towards its implementation in routine clinical practice.

**Trial registration:**

Netherlands Trial Register NL8902, registered on 2020–09-15.

## Background

Hepatocellular carcinoma (HCC) is a frequent cause of cancer death with limited treatment options [1]. Although surgical resection is the most important curative treatment for patients with HCC, it is feasible in only a minority of cases, even when the disease is confined to the liver [1, 2]. Resection is frequently precluded due to insufficiency of the ‘future liver remnant’ (FLR), the part that is to remain after resection. To increase the safety of surgical resection, a common strategy is to preoperatively embolize the ipsilateral portal vein [3]. Contralateral shunting of portal blood induces hypertrophy of the FLR that allows safer partial hepatectomy [3]. However, hypertrophy induction may fail especially in patients with low FLR function [4]. In addition, portal vein embolization does not confer tumour control, which is especially disadvantageous in patients with HCC given the lack of effective neoadjuvant therapy. A number of studies have demonstrated accelerated progression of existing tumours after portal vein embolization [5–10]. Therefore, techniques to induce FLR growth that simultaneously confer ipsilateral tumour control may benefit patients.

Radioembolization, an intrahepatic radiation treatment for liver tumours, is a promising candidate technique. Radioembolization involves intra-arterial infusion of yttrium-90 (^90^Y) (SIR-Spheres®, Sirtex or TheraSphere™, Boston Scientific) or holmium-166 (^166^Ho) (QuiremSpheres®, Quirem Medical) loaded microspheres to the liver. Patients who received unilobar treatment (i.e., to the right or left hepatic artery) were found to have marked atrophy of the treated lobe and hypertrophy of the non-irradiated lobe [11]. This response to radioembolization has therefore also been coined ‘radiation lobectomy’, but we will use the term ‘unilobar radioembolization’ throughout this paper.

It has been demonstrated that unilobar radioembolization (i) induces slower, but eventually similar or even more pronounced FLR hypertrophy than portal vein embolization; (ii) is safe; and (iii) allows for subsequent resection [11–20]. Resection specimens obtained after radioembolization reveal significant necrosis with complete obliteration of the tumour in 30% of resected specimens [13]. As such, unilobar radioembolization may increase the likelihood of tumour-free resection margins, especially in the case of tumours close to major bilio-vascular structures. Unilobar radioembolization can be used as a ‘test-of-time’: if new lesions are discovered during the FLR hypertrophy phase—for example due to the outgrowth of already existing micro metastases—this prevents unnecessary surgery. Although all phases of the unilobar radioembolization-surgery treatment modality have been studied retrospectively, prospective studies are still lacking.

Currently available microspheres include ^90^Y and ^166^Ho-based microspheres. The characteristics of the radionuclide ^90^Y limit its use as a scout dose to simulate treatment, as it cannot be accurately visualized at low activity. Therefore, the safety scout dose must consist of surrogate particles (namely Technetium (99mTc) albumin aggregated (^99m^Tc-MAA) that differ substantially from ^90^Y microspheres in size, number and shape, lowering their validity as a predictor for microsphere distribution [21]. The newly developed ^166^Ho microspheres have distinctive advantages over the existing ^90^Y microspheres in terms of quantitative nuclear imaging. ^166^Ho can be visualised on both SPECT/CT and MRI, which improves estimation of lung shunting, intrahepatic distribution, and assessment of therapeutic activity [22, 23]. As the treated non-tumourous liver-absorbed dose seems to be of importance in driving FLR response, accurate dosimetry is assumed to be a prerequisite for optimal safety and efficacy in the unilobar radioembolization setting [18].

We previously showed that ^166^Ho radioembolization, with a projected average absorbed dose of up to 60 Gy to the whole liver, was safe for patients with liver metastases [24], and hepatocellular carcinoma [25]. However, the optimal safety dose to the non-tumourous liver tissue of the treated lobe for patients designated for unilobar radioembolization has not been established. Such data is especially important for patients with HCC, who often have vulnerable livers due to chronic liver disease [1, 2]. In this dose escalation study, the safety and efficacy of ^166^Ho unilobar radioembolization will be investigated in patients with hepatocellular carcinoma that are irresectable due to insufficiency of the FLR.

## Methods/design

### Aims and outcomes

The primary objective of this study is to establish the maximum tolerated non-tumourous liver-absorbed dose of ^166^Ho microspheres in patients with HCC who receive unilobar radioembolization as a bridge to resection. The maximum tolerated dose is assessed in terms of the rate of unacceptable dose-limiting toxicities (see paragraph on Design). All grade 3 or higher toxicities (according to the CTCAE version 5.0 criteria) that are possibly, probably or definitively related to unilobar radioembolization are considered a dose-limiting toxicity. Exceptions to this are expected adverse events: grade 3 or higher lymphopenia and liver enzymes (AST/SGOT, ALT/SGPT, GGT, ALP), as well as post-embolization syndrome. Toxicities after unilobar radioembolization will be recorded at 6 weeks, 3, 4.5, 6 and 9 months, or up to three months after surgery. Administration technique related adverse events will not be regarded as dose-limiting toxicity.

Secondary aims include establishing a dose–response relationship between (i) the perfused/treated normal liver-absorbed dose and the FLR response and (ii) the tumour-absorbed dose and the tumour response, to map the safety and feasibility of surgical resection of converted patients, and to assess quality of life during the study procedures. FLR response will be quantified using MRI (volume of the FLR) and hepatobiliary iminodiacetic acid (HIDA) scan (function of the FLR). Tumour response will be assessed using the mRECIST criteria [26]. Dose–response relationships will be measured at baseline and at every post-unilobar radioembolization follow-up. The safety and feasibility of surgical resection will be monitored through the Clavien-Dindo grading system during the post-operative stay and at the follow-up visit three months after surgery [27]. Quality of life of patients will be assessed at every visit through questionnaires in the patient’s native language. EORTC QLQ C30, EORTC QLQ HCC and BPI-SF questionnaires and manual will be used for assessment and analysis respectively [28–30].

As a last secondary aim, a biobank will be generated of resected liver specimens and blood samples for future analyses of therapy surviving cancer cells.

### Design

**Fig. 1:**
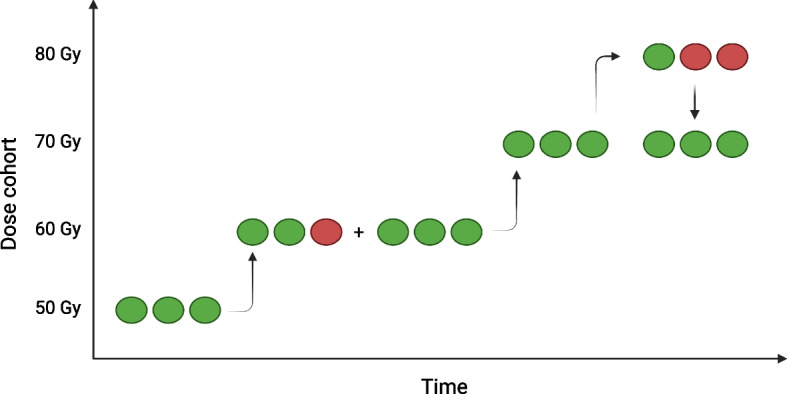
Dose escalation example. Green circles represent patients that did not experience a dose-limiting toxicity at a given dose. Red circles represent patients that did experience a dose-limiting toxicity. The y-axis represents the dose to the non-tumourous liver tissue. In this example, two patients in the 80 Gy cohort experienced a dose-limiting toxicity, meaning the maximal tolerable dose is found and set to 70 Gy (with 18 patients included). The escalation will halt if no maximal tolerable dose is found after 80 Gy. Note that the last safe cohort will always include six patients

The RALLY study is a multicenter, interventional, non-randomized, open-label and non-comparative study. A classical 3 + 3 dose escalation model with four cohorts will be used, enrolling three patients in each cohort (Fig. 1). The first cohort of patients will receive an amount of radioactivity corresponding to a projected average absorbed dose of 50 Gy to the treated non-tumourous liver parenchyma. The non-tumourous liver-absorbed dose is estimated by means of a ^166^Ho-microsphere scout dose [31]. If none of the patients in the first cohort experience a dose-limiting toxicity, the non-tumourous liver dose will be escalated to 60 Gy in a cohort of three new patients. If one patient in a cohort experiences a dose-limiting toxicity, the cohort is increased to six patients. If two or more patients in a cohort develop a dose-limiting toxicity, the maximal tolerable dose is reached and further dose escalation will be ceased. Escalation will cease if no dose-limiting toxicity is found in the 80 Gy cohort. As such, a maximum of four cohorts will be recruited. The last cohort will always consist of six patients to confirm safety.

Inclusion of patients in the next cohort will be performed if (i) all patients have received the treatment dose at least six weeks prior and (ii) the Data and Safety Monitoring Board (see Monitoring) has scrutinized the toxicity data and gave permission to proceed.

### Study population

All patients with HCC in whom upfront surgery is precluded because of insufficient function of the FLR will be included. These are patients with HCC and preserved liver function who have nodules larger than 2 segments, and are not suitable candidates for radiofrequency ablation or liver transplantation (for example in the case of cirrhosis). Insufficient FLR function is defined as a HIDA scan clearance of 1.5—2.7%/min/m^2^ [32]. The complete list of inclusion and exclusion criteria are detailed in Supplementary Table S1. Patients will be identified in the respective tumour boards and subsequently discussed in an expert panel prior to inclusion. Informed consent will be taken by trained personnel with a ‘good clinical practice’ certification who have no treatment relationship with the patient. Patients will be included in the University Medical Center Utrecht, Amsterdam University Medical Center, Erasmus Medical Center (all in the Netherlands) and Regina Elena National Cancer Institute (Italy).

### Intervention, study procedures and timeline

The medical device under investigation contains the radionuclide holmium-166, marketed as QuiremSpheres® and QuiremScout®. In contrast to ^90^Y, ^166^Ho emits both gamma-radiation (81 keV) and high-energy (1.81 MeV) beta-particles. The beta-particles are responsible for the therapeutic effect of the device; the gamma-radiation may be used for nuclear imaging purposes (i.e., SPECT/CT). Patients will receive treatment to just one liver lobe (i.e., the left or right branch of the hepatic artery, ± the segment IV artery).

**Fig. 2:**
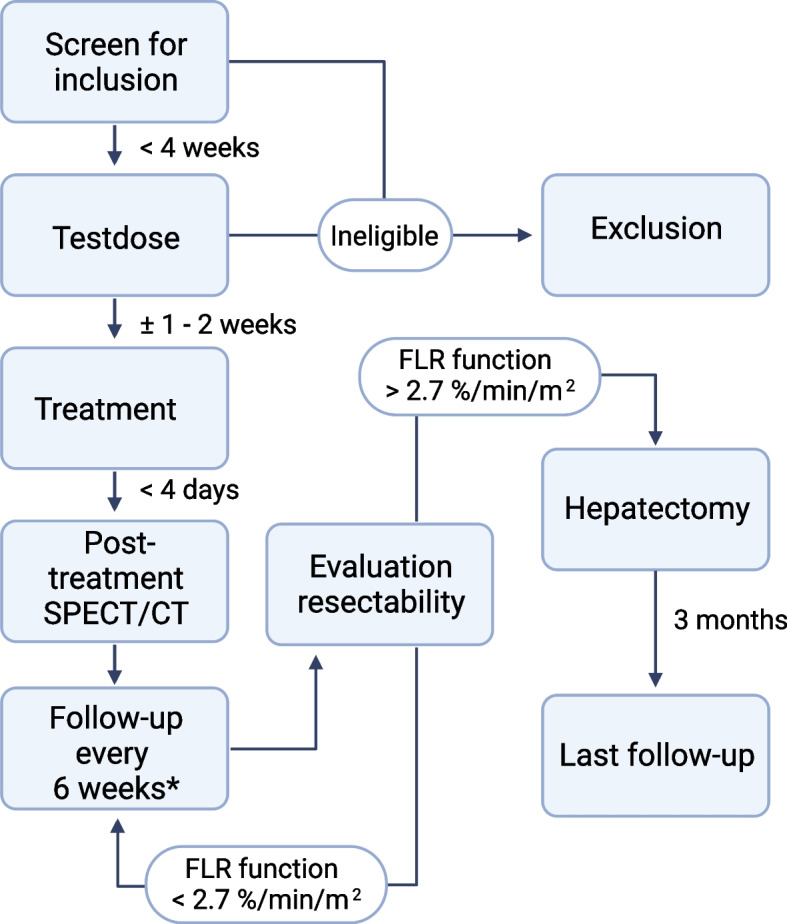
Study flowchart. Eligibility is assessed during the screening visit (patient and tumor characteristics) as well as the scout dose (lung shunting, extrahepatic depositions, etc.). Patients will be considered included once they received the actual treatment dose. The post-treatment follow-up visits serve to map toxicities, as well as to evaluate eligibility for resection. *The exact interval will be at 1.5, 3, 4.5, 6 and 9 months. The last follow-up will be at 9 months following treatment (if not converted) or 3 months after hepatectomy

The study exists of a screening visit, a scout dose, the actual therapeutic dose, the post-unilobar radioembolization follow-up visits, the surgical resection (if patients can proceed to surgery) and the post-surgery follow-up visit. The participant’s timeline is summarized in Fig. 2. Table 1 details the study procedures per visit.

**Table 1:** Study procedures

Procedures	Screen	Scout	Treatment	SPECT/CT	Follow-up^a^	S	Post-S visit^b^
Informed consent	E						
In-/exclusion	E	E					
Demographic data	X						
Physical exam, vital signs and clinical performance status (ECOG)	X	E	X		X	X	X
EORTC QLQ C30, HCC 18, BPI-SF	E	E	E		E	E	E
MRI / CT liver	X				X^c^	X^c^	X^c^
CT thorax	X						
HBS	X				X		
Angiography		E	E^d^				
Scout dose		E					
Therapeutic dose			E				
SPECT/CT + contrast CT		E		E^e^			
Laboratory examination	X	E	X		X	X	X
Monitoring of (S)AE’s + concomitant med		E	X		X	X	X
Pregnancy test		X					

E = extra activity in study context, X = standard care, W = week, M = month, S = Surgery

^a^Exact interval is at 1.5, 3, 4.5, 6 and 9 months. However, post-radioembolization follow up will be discontinued as soon as patients are eligible to undergo surgical resection. ^b^Three months following surgery. ^c^MRI is standard. CT is performed if MRI is contraindicated. ^d^Venogram when standard therapy (PVE) is performed. ^e^Three to four days after the therapeutic treatment, at separate visit in outpatient clinic

### Screening visit

After obtaining informed consent, a screening visit will take place at the outpatient clinic. The study physician checks in- and exclusion criteria and performs a physical examination. Laboratory tests are taken (haematology, coagulation profile and electrolytes, see Table 2). If not already done so, baseline MRI and CT of the liver as well as HIDA scans are performed and assessed for mRECIST criteria and baseline FLR function respectively [26]. All patients are asked to fill out quality of live questionnaires at this and subsequent visits.

**Table 2:** Laboratory examination

Category	Parameter
Haematology	Leukocytes, erythrocytes, haemoglobin (Hb), haematocrit (HCT), mean corpuscular volume (MCV), mean corpuscular haemoglobin (MCH), mean corpuscular haemoglobin concentration (MCHC), and platelet count
Coagulation profile	INR, PT, APTT
Serum chemistry	Sodium, potassium, phosphate, creatinine, total bilirubin, alkaline phosphatase, SGPT/ALT, SGOT/AST, GGT, glucose, albumin, CRP, LDH, alpha-fetoprotein

Total: max 10 ml

### Scout and treatment procedures

Patients who fulfill the selection criteria at the screening visit will first receive a preparatory angiography and scout dose within four weeks. During angiography, the hepatic arterial vasculature is carefully assessed with digital subtraction angiography and C-arm CT. One or multiple injection positions are chosen that will cover the intended liver volume whilst minimizing the chance of activity deposition in non-target organs (e.g., intestines, pancreas). In the unlikely event that a vessel poses a threat to possible extrahepatic deposition of microspheres, it will be coil-embolized.

Then, a scout dose of ^166^Ho-microspheres (i.e,. approximately three million microspheres; 250 MBq) will be administered during the same procedure [30]. Because the gamma-radiation of ^166^Ho is visible even at such low activities, ^166^Ho-scout can be used to predict the intrahepatic distribution of a therapeutic dose of ^166^Ho-microspheres. It can thus be used to estimate the activity needed during the actual treatment to obtain the necessary dose for the specific dose cohort the patient is in (i.e. treated non-tumorous liver-absorbed dose).

After administration of the scout dose, the patient will be transferred to the SPECT/CT and will be subjected to scintigraphy to assess intrahepatic and extrahepatic activity. Both planar imaging of the thorax and abdomen will be performed, as well as SPECT/CT of the abdomen. Images will be evaluated qualitatively and quantitatively. The angiography, scout dose administration and SPECT/CT are performed during a one-day visit.

The following formulas are used to calculate the activity needed during the actual treatment [33]:

$$A={D}_{NTT}*\frac{(TNR*{M}_{T})+{M}_{NTT}}{15.9*\left(1-LSF\right)}$$

where $A$ is the activity in GBq; ${D}_{NTT}$ the desired dose to the ipsilateral non-tumourous liver tissue ($NTT$) (i.e., 50 Gy, 60 Gy, etc.); $TNR$ the tumour-to-non tumour liver tissue ratio; ${M}_{T}$ and ${M}_{NTT}$ the mass of the tumour ($T$) and ipsilateral non-tumourous liver tissue ($NTT$) in kg; and $LSF$ the lung shunt fraction. The lung shunt fraction is calculated as:

$$LSF=\frac{{C}_{lungs}}{{C}_{lungs}+{C}_{liver}}$$

where $C$ indicates the total counts calculated on post-scout SPECT/CT in the lungs and the perfused liver.

Lastly, the tumour-to-non tumour liver tissue liver tissue ratio ($TNR$) can be inferred from the scout distribution using:

$$TNR=\frac{{A}_{T}*{M}_{T}^{-1}}{{A}_{NTT}*{M}_{NTT}^{-1}}$$

where ${A}_{T}$ and ${A}_{NTT}$ is the total activity to the tumour ($T$) and non-tumourous tissue ($NTT$) measured on the post-scout SPECT/CT.

Besides predicting the distribution within the perfuse lobe, the scout dose serves to exclude any extrahepatic deposition during the actual treatment. It is as such a safety procedure. Notably, estimated lung shunting of more than 30 Gy based on the post-scout SPECT/CT is a contraindication for treatment dose. Activity in the falciform ligament, portal lymph nodes and gallbladder is accepted.

If the scout dose does not reveal any contraindications, patients will undergo ^166^Ho unilobar radioembolization treatment within 2 weeks. Treatment will be performed in a similar fashion as the preparatory angiography, with identical injection positions. SPECT/CT will follow in a separate visit three-to-four days after the intervention to quantify dosimetry.

### Post-treatment follow-up

After treatment, patients will be tentatively scheduled for follow-up visits after 1.5, 3, 4.5, 6 and 9 months. The main goal of these follow-up visits is to evaluate the eligibility of the patient to proceed to surgical resection and to map the toxicity and response following ^166^Ho-radioembolization. The study participation for a patient will end if the patient is unable to undergo surgical resection within 9 months. During each visit, laboratory (Table 2) and clinical toxicities will be monitored. Tumour response is assessed using MRI (or CT if contra-indicated) with mRECIST [26]. FLR response is assessed using HIDA and MRI.

### Hepatectomy and post-surgery follow-up

If tumour burden, performance status and FLR (> 2.7%/min/m^2^) allows for resection, the patient will be operated on as soon as possible. Eligibility for resection will be discussed in a multidisciplinary tumour board. The patient undergoes general anesthesia and the abdomen will be explored to rule out extrahepatic disease. The extent of the hepatectomy will be determined intraoperatively with the aim of achieving R0 resection. Directly following resection, surgical specimens of both tumour- and non-tumour tissue will be processed for routine histological evaluation as well as inclusion in the RALLY biobank (see heading ‘Biobank’). After resection, patients will receive a last follow-up visit three months after resection during which toxicities, surgical complications (using Clavien-Dindo) and tumour burden will be assessed [27].

### Biobank

Resection specimens will be fresh-frozen and formalin-fixed paraffin embedded for future molecular analyses. A blood sample (20 mL maximum) will be drawn from the venous line during the surgical procedure. This will be used as a genome reference. The processed body material is stored in the designated RALLY biobank under the management of the UMC Utrecht (in -80° C freezers or in nitrogen vessels for fresh frozen material). For participating sites, the material is first temporarily stored according to the guidelines of the respective center’s biobank. Afterwards, the specimens will be transported to the UMC Utrecht and stored in the RALLY biobank.

### Statistics and data management

#### Statistical analysis

Descriptive statistics (n, mean, standard deviation, 95-CI, median, minimum and maximum) will be calculated for each quantitative variable; frequency counts by category will be made for each qualitative variable. Two sets of study data will be evaluated: the primary endpoint will be evaluated in the Full Analysis Set. The Full Analysis Set is defined as the set of data generated from the included patients who received at least the treatment dose. The secondary endpoints will be evaluated in both Full Analysis Set and Per Protocol Set. The Per Protocol Set is defined as the set of data generated from the included patients who complied with the protocol. A linear mixed effects regression model will be used to evaluate the relationship between absorbed dose- and studied effect. An analysis of variance method with a Chi-square test will be used to compare nested models and generate p-values for the fixed effects. The explained variance by the model, with and without random intercept, will be assessed with R^2^ for mixed effects models.

#### Sample size

This study is designed with four dose cohorts, starting at 50 Gy and going up to 80 Gy. The last cohort will consist of at least six patients. This means that the maximum number of patients (i.e., if in each cohort one dose-limiting toxicity occurs, see Design for details) will be (3 + 3) × 4 = 24 patients. The minimum number of patients will be two, which is when two patients in the first (50 Gy) cohort experience a dose-limiting toxicity. If during the entire study no patient experiences a dose-limiting toxicity, the number of patients will be 3 × 4 + 3 = 15.

#### Data management

Data is collected using the secure online Electronic Data Capture application Castor (Castor EDC). The source data is found in the Electronic Health Record of each respective center. Names of patients and their patient number will be replaced by a computer-generated study code for all study related procedures. A separate file with patient number and specific code will be produced for the biobank. Outcome variables will be analyzed by physicians within the Sponsor’s institute who are not directly involved in the conduct of the study.

#### Monitoring

The External Data and Safety Management Board (EDSMB) safeguards the interests of the trial participants, assesses the safety and efficacy of the interventions during the study, and monitors the overall conduct. The EDSMB will evaluate the toxicity data every three patients. All collected data is monitored by an independent, external data monitor. Severe adverse (device) events will be reported to the Medical Ethical Committee of the University Medical Center Utrecht and the EDSMB.

## Discussion

HCC requires a multidisciplinary approach involving specialists in hepatology, oncology, interventional radiology, nuclear medicine, pathology and surgery. The RALLY study aims to assess the safety of unilobar ^166^Ho unilobar radioembolization as a means of converting patients with insufficient FLR to resectability.

To date, numerous studies investigated the outcomes of unilobar radioembolization [11–20]. Albeit retrospectively, these studies reveal the safety and efficacy of unilobar radioembolization in achieving ipsilateral tumour control and contralateral hypertrophy and indicate that surgical resection of radiated liver lobes is safe and technically feasible. Sufficient FLR response can be expected three to five months following unilobar radioembolization. Virtually all studies base FLR sufficiency on its volume relative to total liver volume. However, assessing FLR function may better predict the risk of post-hepatectomy liver failure [34]. We recently reported favorable surgical outcomes in a set of patients where HIDA scan was routinely used to assess FLR sufficiency [19]. The RALLY study will therefore determine the sufficiency of the FLR with a HIDA scan, using a cutoff of 2.7%/min/m^2^ [32].

**Fig. 3:**
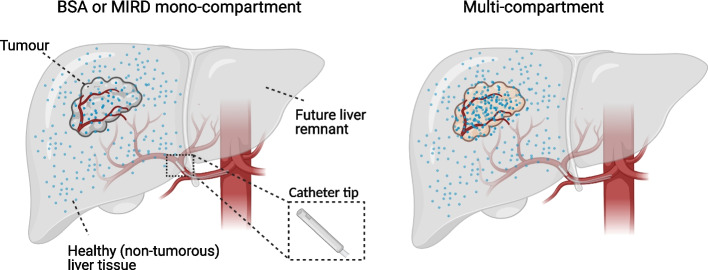
Activity planning models. While the BSA and mono-compartment models assume homogenous distribution within the perfused part of the liver (indicated in the picture with equal distribution of blue microspheres in tumour and non-tumourous liver tissue) the multi-compartment model postulates three compartments with different activity uptakes: tumour, non-tumourous liver tissue and lung tissue (not depicted). The multi-compartment model permits the selection of a prescribed dose that optimizes the dose to one of the compartments – in the case of the RALLY study this is the non-tumorous liver tissue. The expected activities in each compartment will be based on the distribution of the ^166^Ho scout dose. Non-tumourous liver-absorbed dose is an important factor for safety (primary objective of the RALLY study) as well as FLR response (secondary objective) and tumour-absorbed dose determines tumour response (secondary objective). As such the multi-compartment model increases safety and efficacy. Notice that in the RALLY study, microspheres are administered to one lobe and not to the whole liver

Current activity planning methods are either based on the body surface area (BSA) model or the Medical Internal Radiation Dose (MIRD) mono-compartment model (Fig. 3 left panel) [33, 35]. In the BSA model, the prescribed activity is calculated using the total body surface area and the fractional liver involvement. In the MIRD mono-compartment model, the activity is based solely on the target liver mass. Both methods assume a homogeneous distribution of microspheres between tumourous and non-tumourous liver tissue and thus ignore the actual spatial microsphere distribution. This may lead to over- or under dosing [35–37]. A scientifically more sound method is the so-called multi-compartment model (Fig. 3 right panel). This model factors in the microsphere distribution between three compartments -i.e., tumour-, non-tumourous liver- and lung tissue- by obtaining activity output from post-scout SPECT/CT and planar scintigraphy images. This personalized model more accurately predicts absorbed doses [38].

Using ^166^Ho, multi-compartment dosimetry planning can be optimized further. A small number of ^166^Ho microspheres can be used during the scout dose instead of surrogate ^99m^Tc-MAA particles used prior to ^90^Y treatment. ^99m^Tc-MAA particles have different physical properties than ^90^Y microspheres and may negatively influence predictive power of the scout [21, 38, 39]. Indeed, it was shown that the holmium scout is superior in predicting lung shunt as well as intrahepatic dose distribution [22, 23]. Because destruction of functional non-tumourous liver tissue drives FLR response, accurate dosimetry of non-tumourous liver-absorbed dose is pertinent in the unilobar radioembolization setting [18].

In conclusion, this is the first prospective clinical study to assess the unilobar radioembolization-surgery treatment as a whole. The results will likely impact the clinical management of potentially curative HCC patients.

## Data Availability

Not applicable.

## Abbreviations

BSA: Body surface area
EDSMB: External Data and Safety Management Board
FLR: Future liver remnant
HCC: Hepatocellular carcinoma
HIDA: Hepatobiliary iminodiacetic acid
LSF: Lung shunt fraction
^166^Ho: Holmium-166
MIRT: Medical Internal Radiation Dose
^99m^TC-MAA: Technetium (99mTc) albumin aggregated
^90^Y: Yttrium-90
